# Impact of atrial fibrillation on pulmonary embolism hospitalization: Nationwide analysis

**DOI:** 10.1016/j.ahjo.2024.100465

**Published:** 2024-09-24

**Authors:** Mubarak Hassan Yusuf, Akanimo Anita, Olayiwola Akeem Bolaji, Faridat Moyosore Abdulkarim, Chibuike Daniel Onyejesi, Maryam Yusuf, Utku Ekin, Arham Syed Hazari, Mourad Ismail

**Affiliations:** a Division of Pulmonary and Critical Care, Department of Medicine, St Joseph University Medical Center, Paterson, NJ, USA; b Department of Medicine, Lincoln Medical Center, Bronx, NY, USA; c Division of Cardiology, Department of Medicine, Memorial Sloan Kettering Cancer Center, NY, USA; d Department of Medicine, University of Nigeria Teaching Hospital, Ituku Ozalla, Nigeria; e Department of Family Medicine, Federal Teaching Hospital, Katsina, Katsina State, Nigeria

**Keywords:** Atrial fibrillation, Pulmonary embolism, Hospital outcomes, VTE, Cardiac arrest

## Abstract

**Introduction:**

Atrial fibrillation (AF) is the most common type of arrythmia affecting approximately 1–2 % of the adult population. Patients with an underlying history of atrial fibrillation have a greater chance of developing venous thromboembolism (VTE). Likewise, patients with VTE are at increased risk for AF. There has been conflicting evidence on the prognostic impact of AF in acute pulmonary embolism (PE) patients. The aim of this retrospective cohort study was to estimate the impact of AF on the clinical outcomes of hospitalization for PE.

**Method:**

The 2016–2021 National Inpatient Sample database was searched for adult patients hospitalized with PE with associated history of AF as the principal discharge diagnosis. The primary outcome was inpatient mortality, while the secondary outcomes were length of stay (LOS), total hospital charge (THC), cardiogenic shock, acute respiratory failure, in-hospital cardiac arrest (IHCA). The outcomes were analyzed using multivariable logistic and linear regression analyses.

**Results:**

A total of 1,128,269 patients were admitted for PE, 12.4 % of whom had underlying AF. The AF and non-AF cohorts had a mean age of 73.6 years and 61.6 years, respectively. PE patient with AF had significantly higher mortality compared to non-AF patients with PE (6.05 % vs 2.75 %, adjusted odds ratio of 1.67 [95 % CI 1.56–1.79; *p* < 0.0001]). The PE with AF cohort had increased odds of cardiac arrest, cardiogenic shock, respiratory failure requiring intubation, higher average length of stay (5.66 days vs 4.18 days, *P* < 0.001) and a higher total hospital cost (65,235 vs 50,118, P < 0.001).

**Conclusion:**

AF was associated with increased inpatient mortality and worse clinical outcomes in hospitalization for acute PE.

## 1 Introduction

Pulmonary embolism (PE) typically arises as a complication of deep vein thrombosis (DVT) in the lower extremities, with emboli originating in the pelvic or upper extremity veins, as well as in the right heart chambers [1]. Atrial fibrillation (AF), the most prevalent arrhythmia, can also contribute to thromboembolic complications. However, studies examining the potential relationship between AF and PE have produced conflicting results [[1], [2], [3], [4], [5]]. The pathophysiologic association between PE and AF arises from the fact that PE can elevate right atrial pressure, potentially triggering AF. Conversely, AF can lead to PE either through direct embolization or via a hypercoagulable state, as observed in certain cases of chronic AF [3].

AF has been shown to influence outcomes in patients with cardiovascular diseases such as acute myocardial infarction and acute heart failure [2]. This can be attributed to the hemodynamic changes observed in AF; for instance, an increase in heart rate can worsen ischemia in patients with acute coronary syndrome [2]. The physiologic derangements that occur in AF can also compromise the body's attempts to compensate in cases of PE, including the effect on preload and atrial function [2]. Due to the reduction in cardiac output in PE, the impact of atrial booster function on preload and cardiac hemodynamics becomes more prominent [2,3]. Based on these physiological associations, it is expected that the presence of AF may have prognostic significance in PE patients [2]. Although this is plausible, there is a paucity of data on the effects of atrial fibrillation on clinical outcomes in patients with PE. Few studies have shown no significant effects on clinical outcomes while other studies have shown significant effects on outcomes [2,[5], [6], [7], [8]].

In this study, we aimed to determine whether there was any significant impact of AF on the clinical outcomes of patients admitted with PE. We compared the difference in inpatient mortality between patients with a primary discharge diagnosis of PE with a secondary diagnosis of AF and those without a diagnosis of AF. We also determined the differences in secondary outcomes between patients with AF and patients without AF.

## 2 Methods

### 2.1 Study design and data source

This was a retrospective cohort study of hospitalized adult patients with a diagnosis of PE with and without a secondary diagnosis of AF. The study adhered to the Strengthening the Reporting of Observational Studies in Epidemiology (STROBE) statement [9]. Patient samples were obtained from the Healthcare Cost and Utilization Project (HCUP) National Inpatient Sample (NIS) of the years 2016, 2017, 2019, and 2020. The NIS is the largest available all-payer inpatient database in the U.S. and was created and maintained by the Healthcare Research and Quality Agency; it was designed as a representative sample of acute care inpatient hospitalizations in the country [10]. Using inpatient stay information, the database contains information on approximately 20 % of all discharges from the participating hospitals and is weighted to represent the total inpatient hospitalizations for each year [11]. The database includes each admission's patient-related and hospital-related information.

### 2.2 Participants, eligibility criteria and exposure

**Fig. 1:**
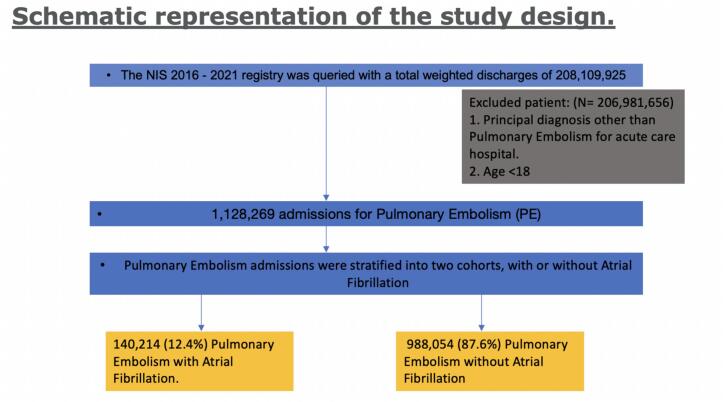
Flow diagram of the study population. This is a schematic representation of the study design; The 2016–2021 NIS registry was queried was a total weighted discharge of 208,109,925. We excluded patients aged <18 years as well as patients with principal diagnosis other than Pulmonary Embolism (PE). 1,128,269 PE admissions were further divided into cohorts with or without Atrial Fibrillation (AF).

The study population consisted of all inpatient hospitalizations recorded in the NIS 2016, 2017, 2018, 2019, 2020 and 2021 datasets for patients aged ≥18 years (selection flowchart in Fig. 1). The study variables included age, sex, race, hospital characteristics, medical comorbidities, and primary and secondary outcomes (outlined later). We used a variety of ICD-10 codes to identify principal and secondary diagnoses (Supplementary Table 1) [12,13]. These specific codes have been employed in previous research studies to identify cases of both PE and AF [[14], [15], [16], [17], [18]]. Patients who were <18 years old or lacked data for any of the variables in the regression analysis were excluded from the study.

### 2.3 Variables

The collected information at the patient level included age, sex, race, expected primary payer, median household income (using the ZIP code), history of hypertension, diabetes mellitus, smoking, hyperlipidemia, myocardial infarction, chronic kidney disease, coronary artery disease, prior CVA, liver disease, pacemaker, anemia, obesity, sarcoidosis, interstitial lung disease, obstructive sleep apnea, chronic anticoagulant use, and chronic obstructive pulmonary disease. Additionally, hospital characteristics were incorporated into the analysis (hospital region, hospital bed size, hospital location and teaching status). A detailed list of the ICD-10 codes used to extract patient information and comorbidities is provided in Supplementary Table 1 [12,13]. The NIS database has been used in prior studies to successfully determine the impact of AF on different groups of patients [15,17,18].

### 2.4 Outcome measures

The primary outcome of the analysis was to compare the difference in inpatient mortality between patients who had a primary discharge diagnosis of PE with a secondary diagnosis of AF and those without a diagnosis of AF. The secondary outcomes were length of stay (LOS), total hospital charge (THC), cardiogenic shock, acute respiratory failure, in-hospital cardiac arrest (IHCA), need for invasive mechanical ventilation, need for thrombolytic therapy, need for EKOS, and need for ECMO.

### 2.5 Statistical analysis

We analyzed the data using Stata/BE software, version 17.0. This technology makes it possible to conduct studies and provide impartial, nationally representative data, variance estimates, and *P* values. For national estimates, analysis was performed using weighted samples in accordance with HCUP standards for the use of the NIS database. Categorical variables (proportions) were compared using the Pearson chi-square test, and continuous variables (means ± SDsSDs) were compared with Student's *t-*test. In the process of generating outcomes, multivariate regression analysis was used to adjust for possible confounders, including age, sex, race, expected primary payer, median household income, past medical history of hypertension, diabetes mellitus, smoking, hyperlipidemia, myocardial infarction, chronic kidney disease, coronary artery disease, prior CVA, liver disease, pacemaker, anemia, obesity, sarcoidosis, interstitial lung disease, obstructive sleep apnea, frailty, pulmonary hypertension, bleeding disorder, history of malignancy, CHA2DVASc score, long-term anticoagulant use and chronic obstructive pulmonary disease. All *P* values were two-sided, with a statistical significance threshold of <0.05.

### 2.6 Ethical considerations

Since the NIS provides de-identified patient data and is openly accessible, this study does not require institutional review board approval, as there are no patient, state-level, or hospital identifiers involved. Consequently, the need for consent to participate is also waived [19].

## 3 Results

### 3.1 Participant characteristics

There were >208 million discharges included in the combined 2016–2021 NIS dataset. A total of 1,128,269 hospitalizations were for adult patients with a principal diagnosis of PE with an ICD-10 code, and 140,215 (12.4 %) of these hospitalizations had AF as a secondary diagnosis. Table 1 displays the characteristics of PE-related hospitalizations with and without coexisting AF.

**Table 1:** Baseline patient and hospital characteristics of patients hospitalized for pulmonary embolism.

Variable	Overall %	PE w/o AF %	PE w AF %	*p*-Value
	*N* = 1,128,269	*N* = 988,054 (87.6)	*N* = 140,215(12.4)	
Patient's characteristics				
Mean age, in years	63.1	61.6	73.6	<0.001
Gender		%	%	<0.001
Female	51.69	52.43	46.45	
Male	48.31	47.57	53.55	
Racial distribution	%	%	%	<0.001
White	72.66	71.33	81.94	
Black	19.82	20.87	12.47	
Hispanic	6.06	6.31	4.3	
Asian/pacific islander	1.05	1.06	0.96	
Native americans	0.41	0.42	0.33	
Insurance type				<0.001
Medicaid	53.83	50.55	76.74	
Medicare	12.53	13.5	5.79	
Private	29.5	31.49	15.6	
Uninsured	4.14	4.47	1.86	
Charlson comorbidity index score	%	%	%	<0.001
0	29.67	31.91	13.86	
1	23.3	23.7	20.53	
2	16.78	16.32	19.97	
≥3	30.26	28.07	45.64	
Median annual income, us$				<0.001
1–43,999	28.68	29.02	26.33	
44,000–55,999	26.55	26.47	27.1	
56,000–73,999	24.6	24.46	25.52	
≥74,000	20.17	20.05	21.04	
Hospital characteristics				
Hospital region				<0.001
Northeast	18.08	18.17	17.51	
Midwest	25.11	25.02	25.74	
South	38.93	39.13	37.5	
West	17.88	17.68	19.26	
Hospital bed size				0.0751
Small	21.9	22	21.21	
Medium	29.27	29.23	29.59	
Large	48.82	48.77	49.2	
Hospital location				0.6889
Rural location	9.38	9.36	9.51	
Urban location	90.62	90.64	90.49	
Hospital teaching status				0.2048
Non-teaching hospital	30.76	30.7	31.16	
Teaching hospital	69.24	69.3	68.84	
Comorbidities				
Hyperlipidemia	37.12	35.68	47.24	<0.001
Previous MI	5.08	4.61	8.41	<0.001
Previous PCI	0.43	0.38	0.77	<0.001
History of CABG	3.09	2.61	6.47	<0.001
Previous pacemaker	1.82	1.12	6.22	<0.001
COPD	17.77	16.48	26.89	<0.001
Coronary artery disease	0.54	0.48	0.94	<0.001
Previous stroke	0.61	0.55	1.08	<0.001
PVD	2.02	1.79	3.64	<0.001
Hypothyroid	12.7	12.19	16.25	<0.001
Diabetes mellitus	23.62	23.05	27.65	<0.001
Obesity	26.11	26.6	22.64	<0.001
Congestive heart failure	17.76	14.45	41.02	<0.001
Obstructive sleep apnea	11.05	10.65	13.9	<0.001
Asthma	8.29	8.6	6.07	<0.001
CKD	13.33	12.1	21.94	<0001
Nicotine use	26.11	25.55	30.07	<0.001
Liver disease	5.19	5.09	5.91	<0.001
Oxygen dependence	4.26	3.93	6.59	<0.001
Anemia	23.87	23.25	28.26	<0.001
Hemodialysis dependant	0.97	0.9	1.51	<0.001
Sarcoidosis	0.48	0.49	0.36	0.020
Interstitial lung disease	1.61	1.5	2.4	<0.001
Long-term anticoagulation use	16.25	14.75	26.87	<0.001
Pulmonary hypertension	11.2	10.37	17.2	<0.001
Aspirin use	15.8	14.97	21.68	<0.001
Friality	0.21	0.18	0.42	<0.001
Mean CHA2DVASc		2	3	<0.001
History of malignancy	12.79	12.34	15.99	<0.001
Bleeding disorder	6.49	6.58	5.85	<0.001

*Abbreviations*: CABG, coronary artery bypass graft; CKD, chronic kidney disease; MI, myocardial infarction; PCI, percutaneous coronary intervention; PVD peripheral vascular disease; COPD chronic obstructive pulmonary disease; w, with; w/o, without.

Patients with concurrent PE and AF were older (mean age 73.6 years) than were those with PE without AF (mean age 61.6 years). A total of 46.45 % of the patients in the cohort with PE and AF were female, while 52.43 % were female in the PE without AF cohort. Regarding the racial distribution of PEs with AF compared to PEs without AF, white patients (81.9 % vs 70.33 %), non-Hispanic black patients (12.47 % vs 20.87 %), and Hispanic patients (4.3 % vs 6.31 %) were included. Furthermore, a greater percentage of patients in the PE with AF cohort (76.74 %) than in the PE without AF cohort (50.55 %) were insured with Medicaid.

### 3.2 Primary outcome

**Fig. 2:**
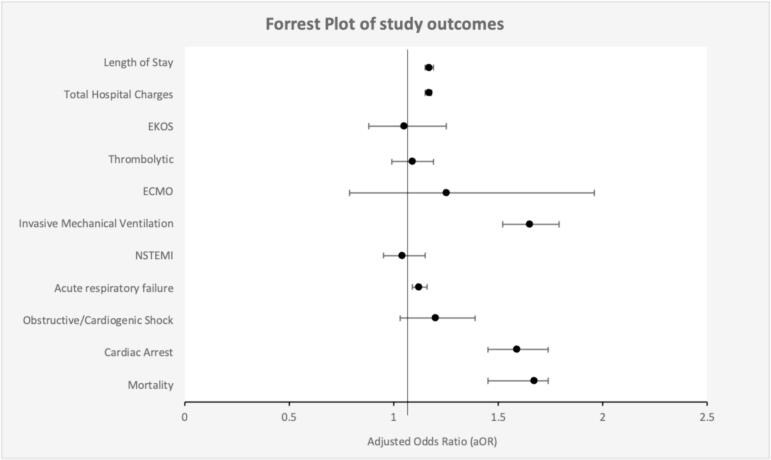
Forest plot representing the in-hospital outcomes of PE patients with AF. A Forrest plot depicting the outcomes of PE patients with AF. Outcomes were ascertained after adjusting for socio-economic factors, hospital status and co-morbidities. The odds of mortality, cardia arrest, cardiogenic shock, acute respiratory failure, mechanical ventilation, NSTEMI were higher among patients with AF. Said cohort with AF incurred higher hospital cost and had longer hospital length of stay. The odds of ECMO, EKOS and thrombolytic therapy were comparable.

There was a total of 35,670 (∼3.16 %) mortality from PE hospitalization, of which 8485 (∼6.05 %) of the deaths occurred with coexisting AF, while 27,185 (2.75 %) occurred without coexisting AF (*P* ≤ 0.001). PE hospitalizations associated with AF exhibited increased in-hospital mortality (2.75 % vs. 6.05 %; aOR, 1.67; 95 % CI, 1.56–1.79; P ≤ 0.001). (Table 2, Fig. 2).

**Table 2:** Clinical outcomes of pulmonary embolism hospitalizations according to atrial fibrillation status.

Outcome	Without AF %	With AF %	aOR (95 % CI)		P-value
Primary outcome					
In-hospital mortality	2.75	6.05	1.67 (1.45–1.74)		<0.001
Secondary outcomes					
Cardiac arrest	1.61	2.88	1.59 (1.451–1.74)		<0.001
Shock	0.69	1.05	1.20 (1.03–1.39)		0.017
Acute respiratory failure	25.7	35.9	1.12 (1.09–1.16)		<0.001
NSTEMI	1.49	2.29	1.04 (0.95–1.15)		0.395
Invasive mechanical ventilation	2.03	3.97	1.65(1.52–1.79)		<0.001
ECMO	0.1	0.12	1.25 (0.79–1.96)		0.324
Thrombolytic	2.46	2.42	1.09 (0.99–1.19)		0.062
Ekos	0.71	0.63	1.05 (0.88–1.25)		0.51
			Adjusted IRR	Adjusted MD	
Length of stay, mean days	4.18	5.66	1.17 (1.15–1.18)	0.82(0.75–0.90)	<0.001
Total hospital charges, mean us$	50,118	65,235	1.17(1.15–1.19)	9390(8214–10,568)	<0.001

*Abbreviations*: aOR, adjusted odds ratio; MD, mean difference; IRR, incidence rate ratio; ECMO, extracoporal membrane oxygenation; NSTEMI, non-st elevation myocardial infarction; CI, confidence interval; us$, united states dollars. Conditions adjusted for include age, sex, race or ethnicity, health insurance, hospital teaching status, Charlson comorbidity index and comorbidities (hyperlipidemia, hypertension, diabetes, chronic kidney disease, chronic liver disease, peripheral vascular disease, prior stroke, long-term anticoagulation use, aspirin use, obesity, obstructive sleep apnea, chronic obstructive pulmonary disease, asthma, interstitial lung disease, fraility, pulmonary hypertension, sarcoidosis, hypothyroidism, electrolyte derangement, congestive heart failure, smoking, oxygen dependence, CHA2DVASc, history of malignancy, bleeding disorder and prior history of myocardial infarction).

### 3.3 Secondary outcomes

**Fig. 3:**
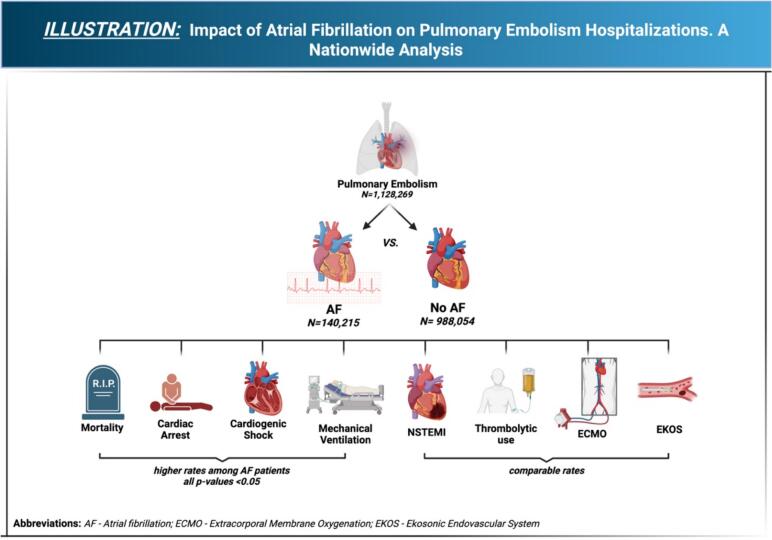
Graphic representation. This graphical illustration represents the in-hospital outcomes of PE hospitalizations with atrial fibrillation when compared to those without AF.

With respect to the secondary outcomes, the PE with AF cohort had increased odds of cardiac arrest (aOR: 1.59; 95 % CI 1.45–1.74; *P* < 0.001), shock (aOR: 1.20; 95 % CI 1.03–1.4; *P* = 0.017), respiratory failure (aOR: 1.13; 95 % CI 1.09–1.16; P < 0.001), requiring intubation (aOR: 1.65; 95 % CI 1.52–1.82; P < 0.001), and an increase in average length of stay (5.66 days vs 4.18 days, adjusted mean difference (aMD) 0.83, 95 % CI 0.75–0.90, P < 0.001) and a higher total hospital cost (65,235 vs 50,118, aMD 9391$, 95 % CI 8214$–10,568$, P < 0.001). There was no difference in AF cohort odds of NSTEMI (aOR: 1.04; 95 % CI 0.95–1.45; *P* = 0.395), needing tPA (aOR: 1.09; 95 % CI 0.99–1.19; *P* = 0.062) or needing EKOS (aOR: 1.05; 95 % CI 0.88–1.25; *P* = 0.51) or needing ECMO (aOR: 1.25; 95 % CI 0.79–1.96; *P* = 0.324) compared to non-AF (Table 2) (Fig. 3/graphic representation).

## 4 Discussion

This was a population-based retrospective study analyzing the impact of AF on the outcomes of patients with PE between 2016 and 2021. The following were our findings: (1) PE patients with AF experienced greater odds of in-hospital mortality than did those without AF. (2) The odds of cardiac arrest, shock, respiratory failure, and invasive mechanical ventilation were greater among PE patients with AF. (3) We noted comparable odds of NSTEMI, ECMO, thrombolytic use and EKOS procedures among the two cohorts. (4) PE patients with AF had longer hospital stays, and they incurred higher costs.

The relationship between AF and PE has not been extensively reported, but controversies regarding the causal association have been identified in the current literature. A large Taiwanese-based retrospective cohort study in 2015 from the Longitudinal Health Insurance Database 2000 (LHID2000) analyzing 11,458 patients with newly diagnosed AF compared to those without AF revealed a greater incidence of PE and deep venous thrombosis (DVT) among patients with AF as well as greater short- and long-term risk [20]. Another long-term study comprising >29,000 subjects investigated the association between AF or venous thromboembolism (VTE) and PE showed higher risk estimates for PE during the first 6 months following a diagnosis of AF than for those without AF [21]. They reported that approximately 15–20 % of PEs could be linked to a thrombus originating from the right atrium in the setting of AF [21]. Furthermore, similar results were replicated in a study by *Hald EM* et al. in 2018*.*, who reported an 11-fold greater risk of PE in AF patients during the first 6 months after diagnosis as well as a steady 72 % increase in risk >/=6 months to 17 years in AF cohorts than in those without AF [22]. Other studies have reported a higher incidence of PE among AF patients [8,23,24]. In contrast, a more recent large-scale Swedish study in 2020, despite reporting a higher incidence of PE among AF patients, revealed no statistically significant association between the two after adjusting for age and concurrent comorbidities [24]; This finding may be explained by the retrospective nature of the study and the lack of a standardized method for classifying AF, as suggested by Ptaszynska-Kopczynska et al. [1]; There appeared to be a stronger association with permanent AF than with paroxysmal AF.

We reported an exponentially greater odd of in-hospital mortality among our PE patients with AF than among those without AF. There are limited studies with controversial reports assessing in-hospital outcomes in this setting [5,6,24]. A 2016 study analyzing 1142 patients with PE, including 207 with baseline AF, reported poorer short- and long-term survival rates among patients with AF than among those without. This difference persisted even after we adjusted for age, sex, Charlson comorbidity index and admission hemodynamic profile [25]. They also reported that among patients with AF, the most common cause of mortality was of cardiovascular etiology, suggesting that the poor prognostic impact of AF is associated with cardiovascular etiology [25]. In 2014, *Barra SNC* et al.*,* investigating a smaller cohort (*n* = 270), reported higher 1-month and 6-month mortality rates among PE patients with AF than among those without AF [7]. Other studies failed to find a statistically significant association between PE and AF on prognosis [5,26]. Furthermore, it remains unclear whether the adverse prognosis observed in patients with AF and PE is attributable to the effects of AF, PE, or a combination of both.

In our study in the same context, PE cohorts with AF had higher rates of cardiac arrest, cardiogenic shock, respiratory failure, and subsequent need for invasive mechanical ventilation. This observation may be indicative of compromised baseline characteristics, including a higher incidence of cardiovascular risk factors such as diabetes, hypertension, hyperlipidemia, or coronary artery disease, as well as overt cardiovascular diseases within this cohort. Additionally, these observation could be associated with unfavorable prognoses commonly linked to individual diagnoses of AF or PE [[27], [28], [29]]. A meta-analysis encompassing 10 studies and a total of 8209 patients analyzing the 12‑lead electrocardiography findings that predict shock in patients with PE revealed greater odds of hemodynamic collapse among AF patients [30]. Furthermore, our findings align with other studies highlighting increased rates of acute myocardial infarction, potentially contributing to the heightened risk observed in our documented outcomes, along with prolonged in-hospital stays and higher healthcare charges among patients with AF [22,31].

According to the European Society of Cardiology, in collaboration with the European Respiratory Society, the management of PE includes anticoagulants, systemic thrombolysis, percutaneous catheter-directed treatments, and surgical embolectomy according to the estimated severity of said illness [32]. Risk scores are also employed in the triage of said patients [32]. These studies do not specifically consider heart rhythm, suggesting that the presence or absence of AF may not significantly affect the choice of therapy. This translates to the comparable rates of EKOS, and thrombolytic therapy reported in our study across cohorts both with and without AF.

### 4.1 Limitations

This study is not without its limitations. The study data were collected from the National Inpatient Sample Database; therefore, we acknowledge that the inherent limitations associated with a retrospective analysis could be apparent. We were unable to account for the severity of illnesses on admission. Despite the use of best practices, errors associated with the use of ICD-10 codes could be replicated. Nevertheless, we believe this paper would be a significant addition to the literature given that the NIS database allows for analysis of a large sample size, resulting in a well-powered study.

## 5 Future directions

Our study's findings highlight the significant impact of atrial fibrillation (AF) on outcomes in patients hospitalized with pulmonary embolism (PE). These results underscore the need for further research to understand and manage this high-risk population better. We propose the following directions for future investigations:

1. Prospective Studies: Large-scale, multi-center prospective cohort studies are needed to confirm our retrospective findings and establish temporal relationships between AF and PE. These studies should track long-term outcomes beyond hospital discharge to provide a more comprehensive understanding of the AF-PE relationship.
2. Mechanistic Investigations: Future research should focus on elucidating the pathophysiological mechanisms underlying the association between AF and worse PE outcomes. This could involve examining hemodynamic changes in AF patients with PE using advanced imaging techniques such as echocardiography and cardiac MRI, with a particular focus on right ventricular function.
3. Risk Stratification: Development and validation of new risk stratification tools that incorporate AF status are crucial. Existing PE severity scores (e.g., PESI, sPESI) should be modified to include AF as a variable, and their performance should be evaluated in prospective studies.
4. Treatment Strategies: It is essential to investigate optimal management strategies for PE patients with AF. This includes comparing the outcomes of different anticoagulation regimens, evaluating the efficacy and safety of catheter-directed therapies, and assessing the role of rhythm control strategies in this population.
5. Subgroup Analyses: Detailed subgroup analyses should be conducted to identify high-risk populations. This includes stratifying by AF type (paroxysmal, persistent, permanent), analyzing outcomes based on prior anticoagulation status, and investigating the impact of AF duration on PE outcomes.
6. Biomarker Studies: Identifying and validating biomarkers that may predict adverse outcomes in PE patients with AF could improve risk assessment and guide treatment decisions.
7. Quality of Life and Long-term Outcomes: Evaluating the impact of AF on quality of life and long-term outcomes in PE survivors through longitudinal studies will provide valuable insights into the overall burden of this comorbidity.
8. Health Economics Research: Cost-effectiveness analyses of different management strategies for PE patients with AF are necessary to inform clinical decision-making and healthcare policy.

By pursuing these research directions, we aim to develop more tailored and effective approaches for managing this high-risk patient population, ultimately leading to improved outcomes and reduced mortality rates for patients with both AF and PE.

## 6 Conclusion

PE is a serious life-threatening VTE that is associated with high mortality and morbidity. AF is the most common cardiac arrythmia and is associated with an increased risk of developing VTE. Our study revealed that AF has a poor prognostic impact on hospitalization for PE including increased rates of in-hospital mortality, cardiac arrest, and increased healthcare resource utilization.

The following is the supplementary data related to this article.

Supplementary Table 1 Supplementary Table 1

## Abbreviations

PE: Pulmonary Embolism
AF: Atrial fibrillation
ECMO: Extracorporeal membrane oxygenation
ICD-10: International Classification Diseases, Tenth
IHCA: in-hospital cardiac arrest
NSTEMI: Non ST elevation myocardial infarction
NIS: Nationwide inpatient sample
LOS: length of stay
THC: Total hospital cost

## Consent for publication

No identifiable patient data or identified individual responses are used in this publication.

## Funding

The authors did not receive support from any organization for the submitted work.

## Statement of ethics

The study was not submitted for research ethics approval as the activities described were conducted as part of the Nationwide Inpatient Sample Database (NIS), which is part of the family of databases and software tools developed for the Healthcare Cost and Utilization Project (HCUP) and uses de-identified data collected from hospitalized patients. Consent was not obtained, given the use of a de-identified database. All the experiments in our study were under the guidelines and agreement regulations of the Agency Healthcare Research and Quality (AHRQ).

Ethical approval and consent were not required as this study was based on publicly available data.

Patient consents were not required as this study was based on publicly available data.

## CRediT authorship contribution statement

**Mubarak Hassan Yusuf:** Writing – original draft, Methodology, Formal analysis, Conceptualization. **Akanimo Anita:** Writing – original draft, Validation. **Olayiwola Akeem Bolaji:** Software, Data curation. **Faridat Moyosore Abdulkarim:** Writing – original draft. **Chibuike Daniel Onyejesi:** Writing – original draft. **Maryam Yusuf:** Visualization. **Utku Ekin:** Writing – review & editing. **Arham Syed Hazari:** Writing – review & editing. **Mourad Ismail:** Supervision.

## Declaration of competing interest

All Authors declare no competing financial or non-financial interest.

## Data Availability

The datasets generated and analyzed during the current study are available in the Healthcare Cost and Utilization Project National Data Registry (https://www.distributor.hcup-us.ahrq.gov/Databases.aspx). This Data Use Agreement (“Agreement”) governs the disclosure and use of data in the HCUP Nationwide Databases from the Healthcare Cost and Utilization Project (HCUP), which the Agency maintains for Healthcare Research and Quality (AHRQ). Accordingly, HCUP Databases may only be released in “limited data set” form, as the Privacy Rule defines that term, 45C.F.R. § 164.514(e). In addition, AHRQ classifies HCUP data as protected health information under the HIPAA Privacy Rule, 45C.F.R. § 160.103. The datasets generated and analyzed during the current study are not publicly available except for the corresponding author who purchased the data and signed the HCUP Data Use agreement training. Researchers should readily be able to publicly purchase the same databases we did to conduct research. Contact information for further guidance on https://www.hcup-us.ahrq.gov/.
