# Supplementary material for: Impact of atrial fibrillation on pulmonary embolism hospitalization: Nationwide analysis
Source: Am Heart J Plus. 2024 Sep 24;46:100465. doi: 10.1016/j.ahjo.2024.100465 (PMC11470505; doi:10.1016/j.ahjo.2024.100465)
Supplement: Supplementary Table 1 [file mmc1.docx]

**Supplementary Table** 1

| Comorbidities | ICD 10 codes |
| --- | --- |
| Pulmonary embolism | I26 |
| Atrial fibrillation | I480 I481 I482 I4891 |
| Hypertension | I10 |
| Diabetes mellitus | E11 |
| Smoking history | 23.5 |
| Hyperlipidemia | E78 |
| Old mi | I25.2 |
| Chronic kidney disease | N18 |
| Coronary artery disease | I65.2 |
| Old pci | Z98.61 |
| Old cabg | Z95.1 |
| Old pacemaker | Z95.0 |
| Aicd | Z95.810 |
| Electroltye abnormalities | E87.0 E87.1 E87.2 E87.3 E87.4 E87.5 E87.6 |
| Peripheral vessel disease | I73.9 |
| Hypothyroid | E03 |
| Obesity | E66.0 E66.01 E66.09 E66.1 E66.2 E66.8 E66.9 |
| Dialysis dependent | Z992 |
| Obstructive sleep apnea | G4733 |
| Sarcoidosis | D86 |
| Pulmonary hypertension | I270, I272 |
| Interstitial lung disease | J84 |
| Copd | J449, J439, J438, J42, J41 |
| Frailty | R54 |
| Aspirin use | Z7982, Z7902 |
| Anticoagulation use | Z901 |
| Congestive heart failure | 10.2 |
| Liver disease | K70 K71 K72 K73 K74 K75 K76 K77 |
| Oxygen dependence | Z99.81 |
| Anemia | D50 D51 D52 D53 D55 D56 D57 D58 D59 D60 D61 D62 D63 D64 |
| Cardiac arrest | I46 |
| Shock | R578 |
| Nstemi | I214 |
| Invasive mechanical ventilation | 0BH17EZ |
| Respiratory failure | J96.00 J96.01 J96.02 J96.20 J96.21 J962.2 J96.90 J96.91 J96.92 |
| Ecmo | 5A1522F 5A1522G 5A1522H |
| Thrombolytic | 3E03317 |
| Bleeding Disorder | D68 |
| History of Malignancy | Z85 |
